# Neuroprotective effects of physical exercise in mouse model of Alzheimer's disease 5Xfad

**DOI:** 10.1002/alz70855_104118

**Published:** 2025-12-23

**Authors:** Anallely Pantoja, Sofía Díaz‐Miranda, Humberto Martínez‐Orozco, Azucena Aguilar‐Vázquez, Jesús Andrade‐Guerrero, Ana Gabriela Hernández‐Puga, Luis Oskar Soto‐Rojas

**Affiliations:** ^1^ UAQ, Querétaro, QA, Mexico

## Abstract

**Background:**

Alzheimer's disease (AD) is the most common neurodegenerative disorder, characterized by the deterioration of executive and cognitive functions. In its late stages, it may also present with significant motor impairment. Its histopathology includes aggregates of Tau protein and beta‐amyloid (ßA) peptide, the latter forming amyloid plaques associated with neuroinflammation, oxidative stress, and neurodegeneration. Currently, there is no curative treatment for AD. However, physical exercise has demonstrated neuroprotective effects at both cognitive and molecular levels in patients and animal models. Nonetheless, its effectiveness varies due to the lack of a standard protocol that can be clinically translated.

**Method:**

In this project, we used the 5xFAD transgenic model, which includes five mutations in the amyloid precursor protein (*APP*) and presenilin genes (*PSEN1 and PSEN2*), associated with ßA synthesis. We evaluated the neuroprotective effects of a physical exercise protocol ( The exercise is performed with a frequency of 5 days a week for 30 minutes at a speed of 10m/min) n male 5xFAD mice, starting at 3 months of age. At the end of the protocol, motor activity (open field test), spatial memory (Y‐maze test), and neuropathological markers were analyzed through immunohistochemistry.

**Result:**

Our results showed significant improvements in spatial memory and motor skills following the intervention. Interestingly, similar improvements were also observed in control individuals, suggesting that physical exercise benefits motor and memory functions even under physiological conditions. Although no changes were observed in ßA immunostaining density, a reduction in the number of diffuse amyloid plaques in the CA1 region of the hippocampus was evident.

**Conclusion:**

These findings suggest that the evaluated exercise protocol could be a promising non‐pharmacological complementary strategy for AD treatment, highlighting its potential to enhance cognitive and motor functions in healthy conditions.

